# Hydrazone-schiff base derivatives of 4-(tert-butyl)benzoic acid as potent enzyme inhibitors: *In vitro* α-amylase, α-glucosidase, tyrosinase inhibition and computational studies

**DOI:** 10.1371/journal.pone.0348140

**Published:** 2026-05-11

**Authors:** Imen Zghab, Sajjad Ahmad, Imtiaz Ahmad, Aftab Alam, Shah Mulk, Zainab Aziz, Masroor Kamal, Ferjeni Zouidi, Abdulrahman S. Alharbi, Ahmed A. Elhenawy, Momin Khan

**Affiliations:** 1 Department of Physical Sciences, Chemistry Division, College of Science, Jazan University, Jazan, Kingdom of Saudi Arabia; 2 Department of Chemistry, Abdul Wali Khan University, Mardan, Pakistan; 3 Programa de Pós-Graduação em Bioquímica e Bioprospecção, Universidade Federal de Pelotas, Campus Universitário Capão do Leão S/N, Pelotas, Brazil; 4 Department of Chemistry, Rawalpindi Women University, Rawalpindi, Pakistan; 5 Department of Biological Sciences, Islamic International University, Islamabad, Pakistan; 6 Institute of Chemical Sciences, University of Peshawar, Peshawar, Khyber Pakhtunkhwa, Pakistan; 7 College of Bionic Science and Agriculture Engineering, Jilin University, Changchun, People’s Republic of China; 8 Faculty of Science and Arts, Muhayil Asser, King Khalid University, Abha, Saudi Arabia; 9 Department of Chemistry, College of Science and Humanities-Dawadmi, Shaqra University, Shaqra, Saudi Arabia; 10 Chemistry Department, Faculty of Science, Al-Azhar University, Nasr City, Cairo, Egypt; Helwan University, EGYPT

## Abstract

Compounds containing an azomethine functional group are well recognized for their enzyme inhibitory potential. In this study, a series of hydrazone-Schiff base derivatives from 4-(tert-butyl)benzoic acid was synthesized and evaluated for their inhibitory activity against α-amylase, α-glucosidase, and tyrosinase. assays demonstrated pronounced multi-target inhibition. Against α-amylase, compound **2l**, bearing a para-nitro substituent, exhibited strong activity with an IC_50_ of 2.72 ± 0.09 µM, significantly outperforming the reference acarbose (IC_50_ = 16.06 ± 0.05 µM; p < 0.001). Similarly, compound **2l** showed potent α-glucosidase inhibition (IC_50_ of 3.96 ± 0.21 µM), which was markedly superior to acarbose (IC_50_ = 16.65 ± 0.07 µM; p < 0.001). In the tyrosinase inhibition assay, the dimethoxy-substituted derivative **2r** emerged as the most active compound, with an IC_50_ of 5.61 ± 0.03 µM, approximately threefold more potent than kojic acid (IC_50_ = 15.29 ± 1.04 µM; p < 0.001). Molecular docking studies against α-amylase, α-glucosidase, and tyrosinase (PDB IDs: 3BAJ, 5NN5, and 5M8Q, respectively) revealed favorable binding energies ranging from −5.2 to −5.8 kcal/mol and highlighted key interactions with catalytic residues. Density functional theory (DFT) and molecular electrostatic potential (MEP) analyses provided an electronic basis for the observed structure-activity relationships, indicating that enhanced electrophilicity favors glycosidase inhibition, whereas increased nucleophilicity contributes to tyrosinase inhibition. Overall, these findings identify hydrazone Schiff base derivatives as promising scaffolds for the development of multifunctional enzyme inhibitors, with compounds **2l** and **2r** representing potential lead candidates for further optimization.

## 1. Introduction

Melanin is a vital biological pigment responsible for protecting human skin against ultraviolet radiation and environmental stress. It is synthesized in melanocytes located in the basal layer of the epidermis through a tightly regulated biochemical process known as melanogenesis, in which tyrosinase plays a central role [1,2]. While normal melanin production is essential for skin protection, excessive accumulation can lead to hyperpigmentation disorders such as melasma, freckles, ephelides, and senile lentigines [3]. Melanin is widely distributed in nature and is found in plants, microorganisms, and animals, including human skin and hair [4].

Tyrosinase, also known as polyphenol oxidase (EC 1.14.18.1), is a copper-containing monooxygenase enzyme that catalyzes the rate-limiting steps of melanogenesis [5]. Specifically, it catalyzes the hydroxylation of L-tyrosine to L-3,4-dihydroxyphenylalanine (L-DOPA) and the subsequent oxidation of L-DOPA to dopaquinone [6]. Dopaquinone undergoes further reactions leading to the formation of polymeric melanin pigments [7]. Because of its pivotal role in melanin biosynthesis, tyrosinase has long been considered an important therapeutic target for the treatment of pigmentation disorders, melanoma, and other melanin-related pathologies [8–10]. Several tyrosinase inhibitors, including kojic acid, arbutin, and hydroquinone, have been used in cosmetic and pharmaceutical formulations. However, these agents suffer from limitations such as poor clinical efficacy, cytotoxicity, or potential mutagenicity, highlighting the need for safer and more effective tyrosinase inhibitors [11–14].

Diabetes mellitus (DM) is a chronic metabolic disorder and a major global cause of morbidity and mortality [15]. It is characterized by persistent hyperglycemia resulting from impaired insulin secretion, insulin resistance, or both [16]. Among its subtypes, type II diabetes mellitus (T2DM) accounts for approximately 90% of all cases worldwide [17]. Postprandial hyperglycemia plays a critical role in the progression of T2DM and its associated complications. Enzymes such as α-amylase and α-glucosidase are directly involved in carbohydrate digestion and glucose absorption, making them attractive therapeutic targets for glycemic control [18–20]. Inhibition of these enzymes delays carbohydrate breakdown and reduces postprandial blood glucose levels. Although clinically used α-glucosidase inhibitors such as acarbose, miglitol, and voglibose are effective, their use is frequently associated with gastrointestinal side effects, including bloating and diarrhea [21–24]. Consequently, the development of novel inhibitors with improved efficacy and safety profiles remains an important research objective.

Hydrazone–Schiff bases constitute an important class of compounds in medicinal and synthetic chemistry due to their structural diversity, ease of synthesis, and broad spectrum of biological activities [25,26]. These compounds are typically formed through the condensation of hydrazines or primary amines with carbonyl compounds, resulting in the formation of a characteristic azomethine (-C = N-) linkage [27]. Numerous hydrazone–Schiff base derivatives have been reported to exhibit significant pharmacological properties, including anti-inflammatory, antioxidant, anticancer, antimicrobial, antidiabetic, and enzyme inhibitory activities [28–36]. In addition, their strong metal-chelating ability has expanded their applications in chelation therapy and bioinorganic chemistry [37]. These favorable features make hydrazone Schiff bases valuable scaffolds for rational drug design.

Recent studies have demonstrated the therapeutic potential of hydrazone Schiff base derivatives against various biological targets. For example, fexofenadine-based Schiff bases have been reported as effective urease inhibitors, while polyhydroquinoline-based hydrazone derivatives have shown potent anti-tyrosinase and prolyl oligopeptidase activities [38,39]. [36,40]. [41,42], Other reports have highlighted their antimicrobial, antioxidant, antidiabetic, and neuroprotective properties, further supporting their multifunctional potential [43,44].

Given the interconnected nature of metabolic and dermatological disorders, the development of multifunctional inhibitors capable of targeting multiple enzymes represents a promising therapeutic strategy. In this context, simultaneous inhibition of α-amylase, α-glucosidase, and tyrosinase may provide dual benefits by controlling hyperglycemia and mitigating melanogenesis-related complications associated with diabetes. Based on these considerations, the present study reports the synthesis of hydrazone–Schiff base derivatives derived from 4-(tert-butyl)benzoic acid and their in vitro evaluation as inhibitors of α-amylase, α-glucosidase, and tyrosinase, supported by molecular docking and quantum chemical analyses.

## 2. Materials and methods

### 2.1. General

In the present investigation all chemicals, reagents and solvents were procured from Merck, BDH, TCI, and Sigma Aldrich. Thin layer chromatography was used to know the formation of the compounds, while the final structures were confirmed through ^1^H-NMR operating at 400 MHz and HR-ESI-MS spectrometry [45].

### 2.2. Procedure for the synthesis of compound [1 and 2]

In the first step, 4-(tert-butyl)benzoic acid was dissolved in 10 mL absolute methanol, followed by the addition of concentrated sulfuric acid (H_2_SO_4_) to it, and the mixture was stirred at reflux for 2 hours. The reaction progress was monitored by thin layer chromatography (TLC) utilizing 30% polar solvent system of ethyl acetate: *n*-hexane, 1.5:3.5 mL). Upon the completion, it was poured into ice-cold distilled water and neutralized through sodium bicarbonate solution until sparkle stopped. The resulting mixture was extracted by chloroform (3 × 20 mL). The combined organic layer was dried over anhydrous sodium sulfate followed by reduced pressure using a rotary evaporator to afford the crude methyl ester in good yield **[1]**.

To synthesize 4-(tert-butyl)benzohydrazide **[2]**, the desired compound **[1]** was dissolved in methanol (10 mL), followed by the addition of hydrazine hydrate to it by constant stirring. The reaction mixture was stirred at reflux for 3–4 hours. After the completion, the mixture was cooled at room temperature and poured onto crushed ice. The precipitated hydrazide was filtered, washed with distilled water, and dried under vacuum to yield the pure product **[2]**.

### 2.3. Procedure for the synthesis of *N*-acyl hydrazone derivatives (2a-r)

A series of *N*-acyl hydrazone derivatives of 4-(tert-butyl)benzo hydrazide has been synthesized in good to excellent yields. In a typical reaction, 4-(tert-butyl)benzo hydrazide **[2]** was dissolved in 10 mL methanol containing a catalytic amount of glacial acetic acid. Then, different substituted aromatic aldehydes were added, and the reaction mixture was refluxed for 4–6 hours through constant stirring. The progress of the reaction was checked through thin layer chromatography (TLC) method. After the completion, it was poured into a beaker filled with crushed ice. The resulting precipitate was collected by filtration, washed with excess distilled water and dried under air to afford the pure hydrazone derivatives. These derivatives have been characterized through HRESI-MS and ^1^H-NMR spectroscopy. These compounds have been reported earlier by our group as potential anti-urease agents [45].

### 2.4. Procedure for alpha amylase inhibitory assay

The modified standard procedure with minor change was used to perform the alpha amylase assay and the enzyme used in this assay was porcine pancreatic α-amylase. Total 18 compounds were tested at varied concentration (62.5–1000 μM). Every experiment used an ELISA 96-well plate with a final reaction volume of 220 µl per well. Briefly 220 µl of reaction mixtures (20 µl DMSO + 20 µl sample + 10 µl enzyme + 50 µl phosphate buffer + 20 µl starch + 100 µl DNS) were present in each well and starch was used as substrate. Each well received 50 µl of phosphate buffer with a pH of 6.9 after 20 µl of DMSO and 20 µl of each sample were diluted five times in DMSO. Then 20 µl of starch was incubated at 37 °C for 20 minutes in each well. Then each well received 10 µl of the enzyme following incubation. After adding 100 µl of DNS to each well, the mixture was incubated at 37 °C for 30 minutes. ELISA was used to measure absorbance at 540 nm. The alpha amylase inhibitory action was analyzed based on IC_50_ value. Acarbose was used as reference and every test was conducted duplicate.

### 2.5. Procedure for alpha glucosidase inhibitory assay

Alpha glucosidase assay was executed by using a standard method with little modification. In this series total 18 compounds were evaluated within concentration range of 62.5–1000 μM. In this analysis the enzyme used was yeast α-glucosidase. In all experiments, the final reaction volume was 200 µl in each well of ELISA 96 well plate. Briefly each well contained 200 µl of reaction mixtures (10 µl sample +20 µl enzyme + 130 µl phosphate buffer + 40 µl *p*-nitrophenyl-alpha-D-glucopyranoside). *P*-nitrophenyl-alpha-D glucopyranoside was used as substrate. Each sample was diluted five times in DMSO then 20 µl of alpha glucosidase enzyme were added to each well then 130 µl of phosphate buffer were added to each well whose PH was (6.8). 40 µl of *p*-nitrophenyl-alpha-D-glucopyranoside was added to each well and incubate it for 15 min at 37 °C. Absorbance was measured at 405 nm using microplate reader. The activity of the compounds was expressed as the IC_50_ value. Acarbose was used as standard and all tests were performed duplicate.

### 2.6. Procedure for tyrosinase inhibitory assay

The compounds’ tyrosinase inhibitory activity was assessed using a standard procedure with a few minor adjustments. Eighteen compounds in all were tested at various concentrations (62.5–1000 μM). Agaricus bisporus mushroom tyrosinase was utilized. In a 96-well microplate, 10 μL of the test sample was added to each well after 10 μL of tyrosinase enzyme and 160 μL of phosphate buffer at pH = 6.8 (50 mM) were mixed. After adding 20 μL of L-dopa solutions as substrates to each well and incubating the plate for 20 minutes at 28 °C, the absorbance of the mixtures was measured at 475 nm. The IC_50_ value was used to express the compounds’ activity. Kojic acid was used as reference drug and every test was run twice.

### 2.7. Computational studies

#### 2.7.1. Quantum chemical calculations (TD-DFT) for reactivity descriptors.

To clarify the chemical reactivity and electronic properties of the synthesized *N*-acyl hydrazone derivatives, quantum chemical calculations were carried out by TD-DFT. All calculations were conducted with the WEbMO package. The ground-state molecular geometries of most active ligands **(2a, 2c, 2d, 2f, 2j, 2k, 2l, 2m** and **2r)** were fully optimized without any symmetry constraints through the hybrid B3LYP functional [46,47] and the 6–311 + G(d,p) basis set. The energies of the FMOs, namely the HOMO and LUMO, were extracted from the TD-DFT calculations. These energies were used to calculate global chemical reactivity descriptors using the following formulae [48–50]. These descriptors provide profound insights into the molecular stability, reactivity, and charge transfer capabilities, which are crucial for understanding the inhibitory potential of the ligands.

#### 2.7.2. Molecular docking studies.

The crystal structures of co-crystallized enzymes were retrieved from the Protein Data Bank (www.rcsb.org). The protein structure was prepared using AutoDock Tools (ADT) [51]. The crystal structures of the enzymes were retrieved from the Protein Data Bank (PDB) with the following IDs: α-amylase (PDB ID: 3BAJ), α-glucosidase (PDB ID: 5NN5), and tyrosinase (PDB ID: 5M8Q). All water molecules and the native ligand were removed. Polar hydrogen atoms were added, and Kollman united atom charges were assigned to the protein. The three-dimensional structures of the most active synthesized compounds and the standard inhibitors were energetically minimized. The optimized geometries were then used for docking studies. Gasteiger charges were assigned, and non-polar hydrogen atoms were merged. The grid box was centered on the catalytic active sites with dimensions of 40 × 40 × 40 Å³ and a grid point spacing of 0.375 Å to ensure complete flexibility for ligand binding. The exhaustiveness parameter was set to 100 to obtain a comprehensive search of the conformational space. For each ligand, ten independent docking runs were performed, and the conformation with the most favorable (most negative) binding affinity (ΔG, kcal/mol) was selected for further analysis of the protein-ligand interactions. The resulting docking poses were visualized and analyzed using BIOVIA Discovery Studio Visualizer [52] and PyMOL Molecular Graphics System [53]. Key interactions, including hydrogen bonds, hydrophobic interactions, halogen bonds, and van der Waals contacts, were meticulously identified and documented. The binding energy values for each ligand were correlated with their experimental half-maximal inhibitory concentration (IC_50_) values to establish a structure-activity relationship.

## 3. Results

### 3.1. Chemistry

In search of excellent bioactive agents, a series of hydrazone Schiff base derivatives have been synthesized by multistep reactions. In the first step, 4-(*tert*-butyl)benzoic acid was stirred with sulfuric acid in absolute methanol at reflux condition for 2 hours to get the ester **[1]** of it. Secondly, the ester **[1]** was refluxed with excess of hydrazine hydrate in methanol solvent for three to four hours to get the hydrazide **[2]** in good yield. Finally, a number of different substituted benzaldehydes were stirred at refluxed condition with the desired hydrazide **[2]** to get various hydrazone Schiff base derivatives in good to excellent yields **(Scheme-**S1 File). TLC method was employed to know the formation of the compounds, while HR-ESI-MS and ^1^H-NMR spectroscopic methods have been used to confirmed the structures of these compounds. In this study, these hydrazone-Schiff bases were assessed for their *in vitro* anti-diabetic (α-amylase and α-glucosidase) and tyrosinase inhibitory activities. These compounds have been reported as potent urease inhibitors earlier by our group [45].

### 3.2. *In vitro* α-amylase and α-glucosidase inhibitory activities

The synthetic *N*-acyl hydrazone derivatives of 4-(tert-butyl)benzoic acid were assessed for their *in vitro* α-amylase and α-glucosidase inhibitory activities. Among the series, five compounds **2l** (IC_50_ = 2.72 ± 0.09 for amylase and 3.96 ± 0.21 µM for glucosidase), **2j** (IC_50_ = 5.28 ± 0.11 for amylase and 8.26 ± 0.14 µM glucosidase), **2k** (IC_50_ = 12.20 ± 0.22 for amylase and 14.75 ± 0.07 µM glucosidase), **2a** (IC_50_ = 13.80 ± 0.07 for amylase and 14.53 ± 0.12 µM glucosidase), and **2c** (IC_50_ = 15.25 ± 0.08 for amylase and 16.58 ± 0.04 µM glucosidase), showed excellent dual inhibition superior to the standard, acarbose (IC_50_ = 16.06 ± 0.05 for amylase and 16.65 ± 0.07 µM glucosidase). Similarly, seven compounds **2n, 2g, 2q, 2i, 2d, 2p** and **2b** presented good inhibitory activities (IC_50_ = 19.63 ± 0.18 for amylase and 21.50 ± 0.08 µM for glucosidase), (IC_50_ = 22.38 ± 0.17 for amylase and 24.43 ± 0.19 µM for glucosidase), (IC_50_ = 25.43 ± 0.02 for amylase and 25.43 ± 0.02 µM for glucosidase), (IC_50_ = 26.03 ± 0.05 for amylase and 27.35 ± 0.35 µM for glucosidase), (IC_50_ = 32.71 ± 0.02 for amylase and 34.54 ± 0.01 µM for glucosidase), (IC_50_ = 35.77 ± 0.01 for amylase and 37.58 ± 0.35 µM for glucosidase), and (IC_50_ = 37.88 ± 0.25 for amylase and 39.20 ± 0.12 µM for glucosidase) respectively. While the remaining compounds including **2r, 2m, 2h, 2f, 2e** and **2o** attributed moderate to less inhibitions with IC_50_ values ranging from (IC_50_ = 42.24 ± 0.11 for amylase and 43.80 ± 0.41 µM for glucosidase) to (IC_50_ = 59.15 ± 0.13 for amylase and 65.29 ± 1.18 µM for glucosidase) (**Table 1**). In context of α-amylase and α-Glucosidase, among the series, five compounds **2a**, **2c**, **2j**, **2k**, and **2l** showed excellent dual inhibition that was statistically superior to the standard acarbose with p < 0.001 for compounds **2j** and **2l** and p < 0.01 for compound **2a, 2c** and **2k** as shown in S1 and S2 Figs.

**Table 1:** I*n vitro* enzyme inhibitory activities of *N*’-benzylidene-4-(*tert*-butyl)benzo hydrazide derivatives (2a-r).

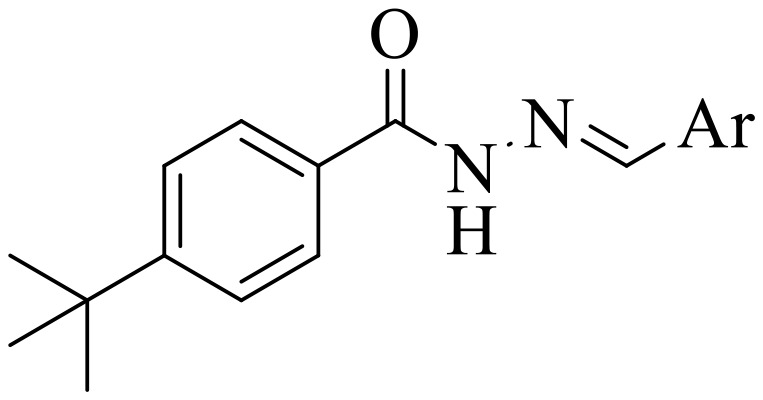				
C. No	Ar	α-amylase	α-Glucosidase	Tyrosinase
		IC_50_ ± SEM *µ*M	IC_50_ ± SEM *µ*M	IC_50_ ± SEM *µ*M
**2a**	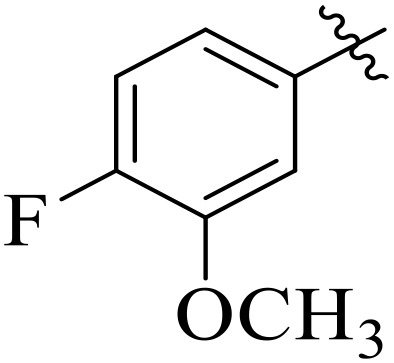	13.80 ± 0.07	14.53 ± 0.12	23.96 ± 0.09
**2b**	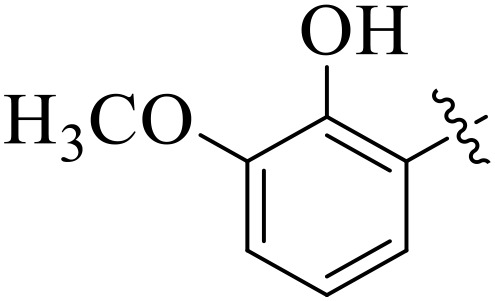	37.88 ± 0.25	39.20 ± 0.12	47.92 ± 0.04
**2c**	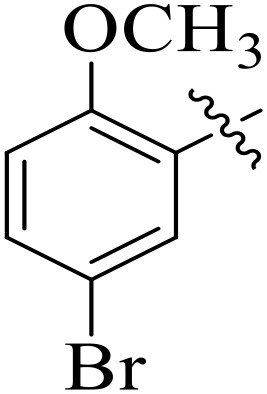	15.25 ± 0.08	16.58 ± 0.04	61.37 ± 0.29
**2d**	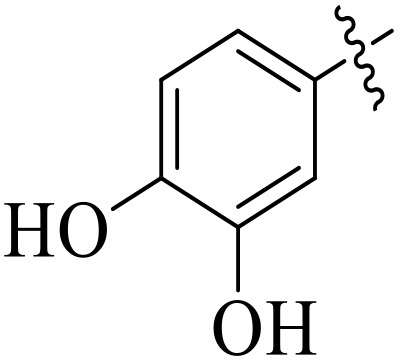	32.71 ± 0.02	34.54 ± 0.01	15.81 ± 0.16
**2e**	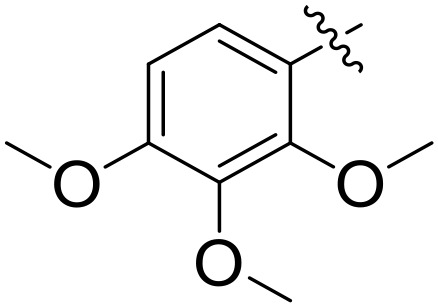	55.67 ± 0.75	57.89 ± 0.17	68.32 ± 0.06
**2f**	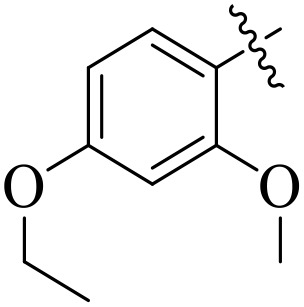	49.80 ± 0.48	53.45 ± 0.27	10.35 ± 0.12
**2g**	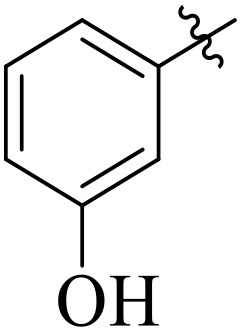	22.38 ± 0.17	24.43 ± 0.19	32.27 ± 0.12
**2h**	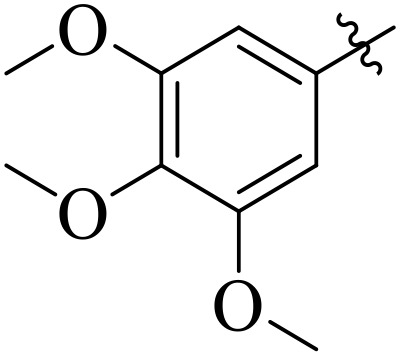	48.69 ± 0.06	52.10 ± 0.27	60.28 ± 0.12
**2i**	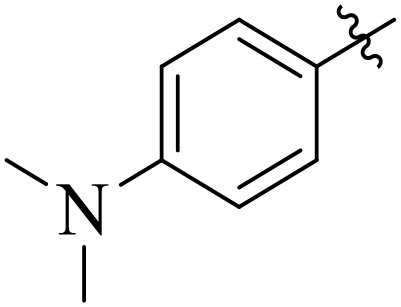	26.03 ± 0.05	27.35 ± 0.35	35.12 ± 0.20
**2j**	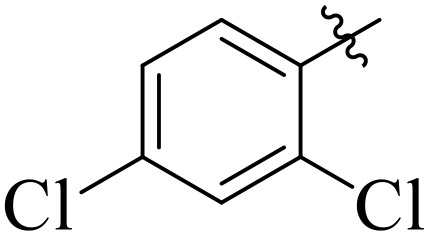	5.28 ± 0.11	8.26 ± 0.14	14.70 ± 0.15
**2k**	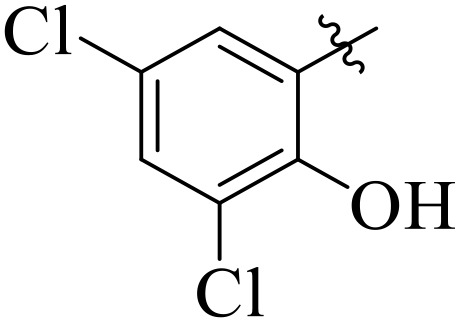	12.20 ± 0.22	14.75 ± 0.07	22.90 ± 0.33
**2l**	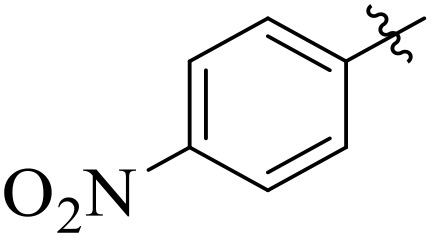	2.72 ± 0.09	3.96 ± 0.21	7.27 ± 0.01
**2m**	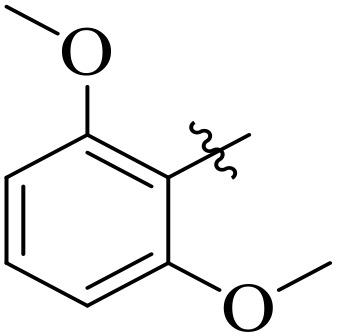	46.86 ± 0.61	48.48 ± 0.31	8.94 ± 0.01
**2n**	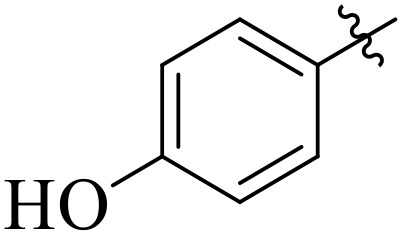	19.63 ± 0.18	21.50 ± 0.08	29.09 ± 0.25
**2o**	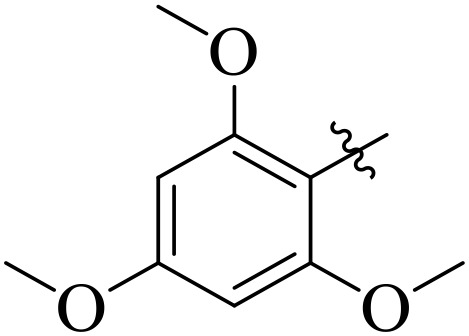	59.15 ± 0.13	65.29 ± 1.18	70.36 ± 1.82
**2p**	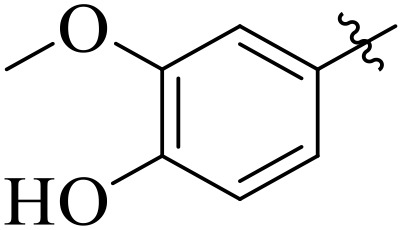	35.77 ± 0.01	37.58 ± 0.35	44.58 ± 0.36
**2q**	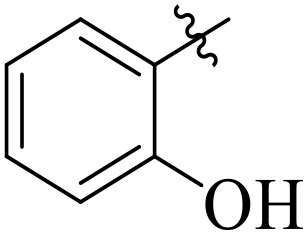	25.43 ± 0.02	25.43 ± 0.02	34.63 ± 0.41
**2r**	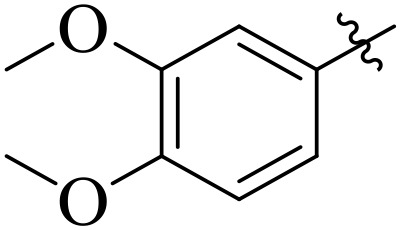	42.24 ± 0.11	43.80 ± 0.41	5.61 ± 0.03
**Standard**	**Acarbose**	**16.06 ± 0.05**	**16.65 ± 0.07**	**------**
	**Kojic Acid**	**------**	**------**	**15.29 ± 1.04**

### 3.3. *In vitro* tyrosinase inhibitory activity

The synthesized compounds have also been screened for their *in vitro* tyrosinase inhibitory activity. Among the series, six compounds **2r** (IC_50_ = 5.61 ± 0.03 µM), **2l** (IC_50_ = 7.27 ± 0.01 µM), **2m** (IC_50_ = 8.94 ± 0.01 µM), **2f** (IC_50_ = 10.35 ± 0.12 µM), **2j** (IC_50_ = 14.70 ± 0.15 µM), and **2d** (IC_50_ = 15.81 ± 0.16 µM) showed excellent tyrosinase inhibitory activity compared with the standard kojic acid. Similar to this, **2k, 2a,** and **2n** displayed good inhibitory activity with IC_50_ values of 22.90 ± 0.33, 23.96 ± 0.09 and 29.09 ± 0.25 µM respectively. However, **2g, 2q, 2i, 2p, 2b, 2h, 2c, 2e,** and **2o** showed moderate to weak activity with IC_50_ value ranging from 32.27 ± 0.12 to 70.36 ± 1.82 µM (**Table 1**). In case of tyrosinase the compounds **2d**, **2f**, **2j**, **2l**, **2m** and **2r** showed highest inhibition and was statistically significant as compared to standard kojic acid with p < 0.001 for compounds **2d**, **2f** and **2j** and p < 0.01 for compounds **2l**, **2m** and **2r (**S3 Fig**).**

## 4. Discussion

### 4.1. Structure activity relationship (SAR)

The synthesized derivatives of 4-(tert-butyl)benzoic acid having different benzaldehydes attributed significant differences in inhibitory activities against α-amylase, α-glucosidase, and tyrosinase, powerfully influenced by both the positional arrangement and electronic nature of substituents.

**Fig 1:**
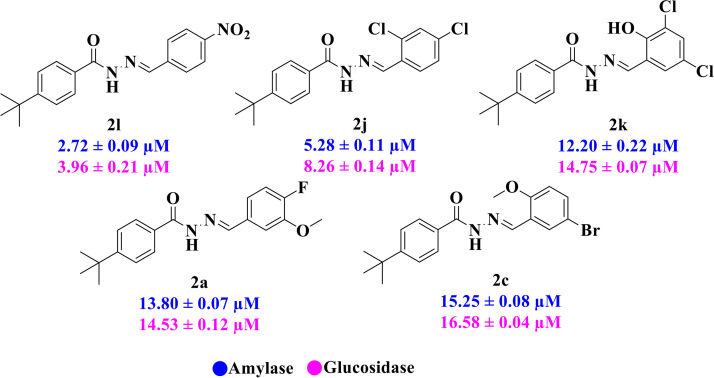
Most active α-amylase and α-glucosidase inhibitors in the series.

For α-amylase as well as α-glucosidase inhibition, compounds having strong electron withdrawing groups showed the most effective activities. Predominantly, compound **2l** bearing a nitro group at the *para* position of the benzene ring showed the lowest IC_50_ values for both the enzymes (2.72 ± 0.09 for amylase and 3.96 ± 0.21 µM for glucosidase), representing that the nitro group strongly increases binding affinity, maybe through favorable hydrogen bonding and amplified polarity with the active site residues. Equally, compounds with halogenated groups that includes **2j** (2,4-dichloro) and **2k** (3,5-dichloro-2-hydroxy) followed with good inhibitory profiles that presented multiple chlorine groups, improve the interaction of enzyme through their increased hydrophobicity and moderate electron withdrawing effects that can support hydrophobic links within the enzyme’s pockets. The position of attached substituents also evidently influenced potency. For example, by comparing compounds **2j** and **2k,** showed that replacing chlorine atoms from *ortho*/*para* to *meta* or adding a hydroxyl group can change the electron distribution and steric profile, dropping enzyme affinity (**Fig 1**). Also, compound **2a**, with a fluorine substituent at *para* and methoxy group at *meta* position also revealed reasonable potency, supporting that a balance of electron donating and withdrawing substituents can still favor enzyme binding when positioned optimally.

In comparison, compounds bearing bulky electron donating groups, like numerous methoxy or alkoxy groups, generally presented condensed potency for α-amylase and α-glucosidase. For example, compound **2e** having 2,3,4-trimethoxy and **2o** with 2,4,6-trimethoxy were amongst the least active that suggest that steric hindrance around the benzene ring and electron donating resonance effects might disturb the optimal binding. Similarly, methoxy comprising compounds like **2h** (3,4,5-trimethoxy) and **2r** (3,4-dimethoxy) showed weaker inhibition, confirming that growing bulk deprived of promising electronic contribution is unfavorable. Compounds containing hydroxyl groups attributed in-between activity, but their effectiveness depended significantly on the hydroxyl position. For instance, compound **2n** (4-hydroxy) was more active compare to **2q** (2-hydroxy), probably due to the hydroxyl group at *para* position that can form direct hydrogen bonds with the active site of the enzymes, whereas an *ortho* hydroxyl substituted compound might contribute in intramolecular hydrogen bonding, restrictive interaction with the target enzyme. The uncertain activity of compound **2g** (3-hydroxy) supports that *meta* hydroxyls contribute less efficiently to binding compared to *para* substituted analogues.

**Fig 2:**
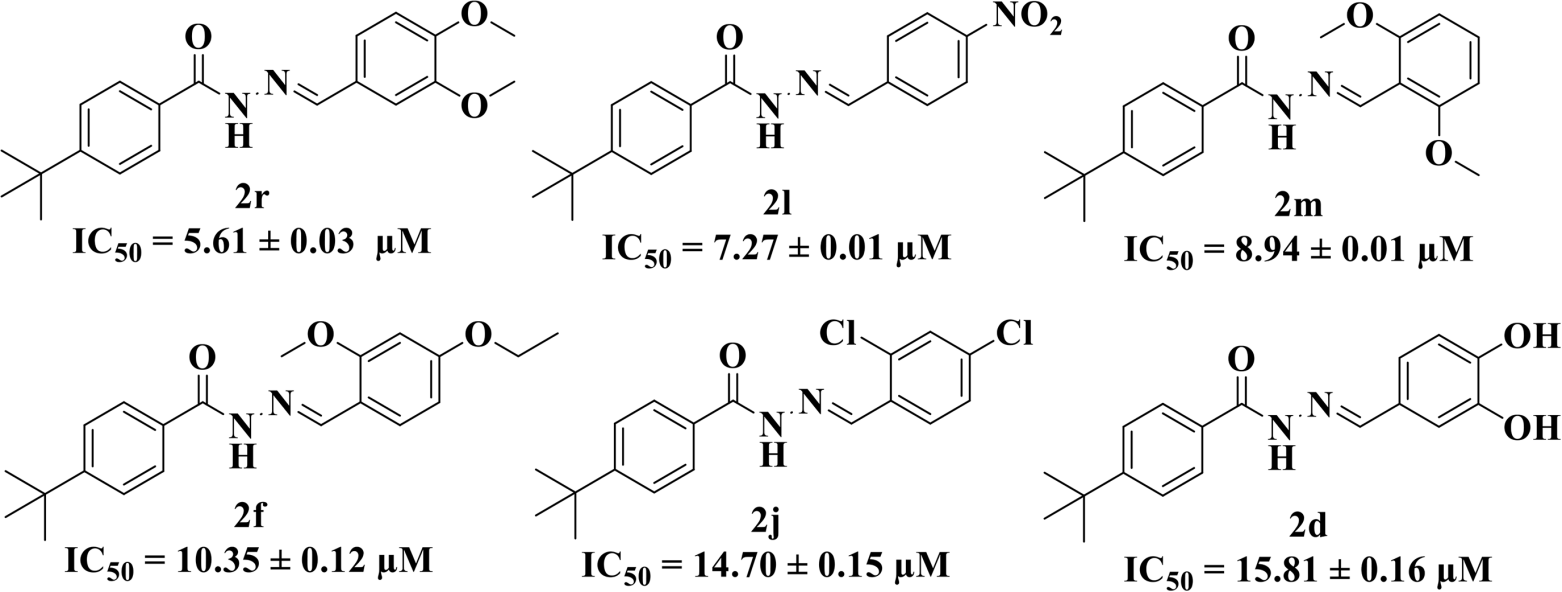
The most active tyrosinase inhibitors in the series.

When comparing tyrosinase inhibitory activity of the compounds, the SAR trends exposed somewhat different design. The most powerful tyrosinase inhibitor was found as compound **2r** having 3,4-dimethoxy group with IC_50_ = 5.61 ± 0.03 µM), beating its comparatively weaker performance against amylase and glucosidase enzymes. This displayed that many methoxy groups attached to the benzene ring at *meta* and *para* positions might better accompany the copper active site of tyrosinase, possibly through chelation interactions and/or π-π stacking. Also, compound **2l** reserved robust tyrosinase inhibition with IC_50_ value 7.27 ± 0.01 µM), showing that the nitro group favors both enzyme classes because of its strong electron withdrawing nature that might improve coordination with the copper ions. Unusually, compound **2m** (2,6-dimethoxy) and **2f** (4-ethoxy-2-methoxy) also presented better tyrosinase inhibition related to their weaker glucosidase and amylase results, suggesting that precise *ortho* methoxy or ethoxy groups might aid binding to tyrosinase by positioning with the active site geometry or facilitating metal chelation. Compounds like **2j** (2,4-dichloro) and **2d** (3,4-dihydroxy) also showed prominent tyrosinase activity, with hydroxyl groups mainly suitable because of their ability to interrelate with the copper ions in the catalytic core of the enzyme’s (**Fig 2**). Comparing compounds **2o** (2,4,6-trimethoxy) and **2e** (2,3,4-trimethoxy) that were already amongst the weakest α-amylase and glucosidase inhibitors, also exhibited the least potent tyrosinase inhibition (IC_50_ > 65 µM). This consistent drop proposes that extreme steric bulk and high electron density from numerous methoxy groups can disturb proper fitting into the tyrosinase active site as well.

Overall, these results specify that robust electron withdrawing substituents like nitro, halogens improve binding for both glycosidic enzymes and tyrosinase by making auspicious electrostatic and hydrogen bonding connections. In the meantime, selective methoxy and hydroxyl groups, especially at *meta* and *para* positions, appear to exclusively benefit tyrosinase inhibition, maybe by interrelating with the copper ions or steadying the enzyme inhibitor complex through π-π communications. Extreme substitution and steric crowding usually decrease potency across all targets because of hindered active site availability and suboptimal binding alignment.

Taken together, this SAR analysis highlights that fine tuning both the electronic nature as well as positional arrangements of the attached substituents is key to scheming potential dual or multi-target enzyme inhibitors with needed biological profiles.

### 4.2. Molecular docking studies of the compounds against α-amylase (PDB ID: 3BAJ)

The inhibition of α-amylase enzyme is well-established therapeutic strategy for the treatment of type-2 diabetes mellitus, as it reduces the rate of carbohydrate digestion and then decreases the postprandial spike in blood glucose levels. To ease the rational design of more effective inhibitors, molecular docking is employed to provide atomic-level understandings into the binding modes and interaction profiles of potential drug candidates. The molecular docking performed against human pancreatic α-amylase (PDB ID: 3BAJ [54,55]). The primary goals are to elucidate the molecular mechanism of inhibition, establish a clear structure-activity relationship (SAR), and compare the binding interactions of these novel compounds with the standard inhibitor, acarbose.

**Fig 3:**
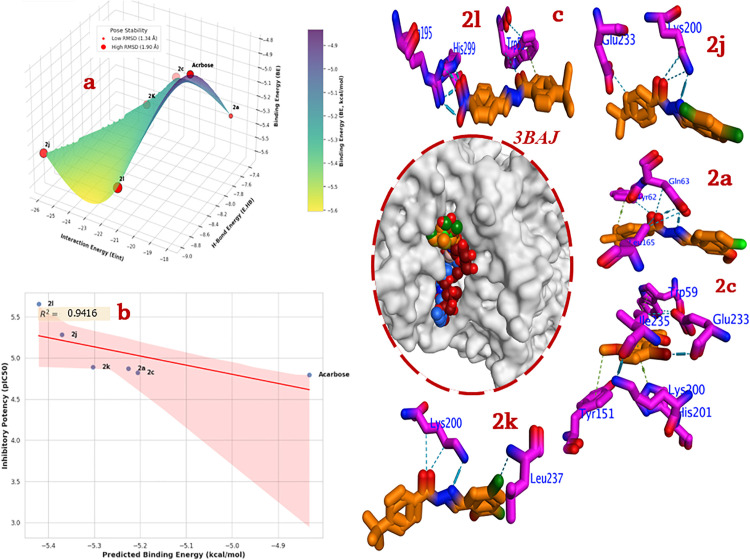
Docking analysis for most active compounds (2l, 2j, 2k, 2a, and 2c) compared to acarbose into 3BAJ; a) Energetic Docking scores, b) Correlation BE against inhibitory potency, c) 3D binding mode, where blue dash represents H-Bond, while green dash is hydrophobic interactions.

The credibility of a molecular docking study hinges on its ability to reproduce and explain experimental observations. A robust correlation between the predicted binding affinity and the experimentally measured biological activity serves as a crucial validation of the computational protocol. As detailed in, an excellent correlation is observed between the calculated binding energy (BE) and the *in vitro* IC_50_ values of the inhibitors. The compound with the highest inhibitory potency, **2l** (IC_50_ = 2.72 ± 0.09 µM), corresponds to the most favorable binding energy (−5.421 kcal/mol). This trend is consistently maintained across the series, with the binding energy becoming progressively less negative as the inhibitory activity decreases, culminating in compound **2c** (BE = −5.205 kcal/mol; IC_50_ = 15.25 ± 0.08 µM). This strong correlation validates the docking methodology and scoring function, confirming that the computational model is a reliable tool for predicting and rationalizing the biological efficacy of these compounds. The plot in **Fig 3a**, and **3b** reveals a strong and clear positive correlation between the predicted binding energy and the experimental inhibitory activity. As the binding energy becomes indicating a more stable and favorable predicted ligand-protein interaction, the IC_50_ value increases, signifying higher measured inhibitory potency. The data points align closely with the linear regression trendline (**Fig 3b**), resulting in a high coefficient of determination (R^2^ = 0.9416). An R^2^ value of this magnitude, being very close to 1.0, indicates that approximately 94% of the variation in the experimental inhibitory activity (IC_50_) can be explained by the variation in the predicted binding energy. This excellent linear association helps as a robust validation of the docking protocol carried out in the current work. It suggests that the scoring function is highly effective in ranking the compounds of this specific class according to their true biological effectiveness. More prominently, it delivers strong evidence that the detailed binding modes and specific molecular connections identified in the docking analysis corroborates the rank-order of these compounds’ inhibitory mode of action.

Acarbose is a pseudo-tetra saccharide that acts as a potent competitive inhibitor of α-amylase by mimicking the structure of the natural substrate. Although a specific binding pose for Acarbose is not depicted, the docking scores provide significant insight. Its high hydrogen bonding energy (E. HB = −8.171 kcal/mol), comparable to the most active compounds, suggests that its mechanism relies on forming an extensive network of hydrogen bonds with the catalytic and substrate-binding residues within the enzyme’s active site, thereby blocking access and preventing catalysis. Its overall binding energy (−4.832 kcal/mol) is less favorable than the synthesized compounds, suggesting that the smaller, more rigid hydrazone derivatives achieve a more efficient combination of enthalpic and entropic contributions to binding in this simulation.

The docking results reveal that all active compounds bind within the enzyme’s catalytic cleft (**Fig 3c**), suggesting a competitive mechanism of inhibition. Their binding is stabilized by a combination of hydrogen bonds and hydrophobic interactions with key active site residues. As the most potent inhibitor, **2l (**BE = −5.421 kcal/mol, IC_50_ = 2.72 ± 0.09 µM) adopts a binding pose that maximizes favorable interactions. The terminal phenyl ring engages in a π-π stacking interaction with the indole ring of Trp59, a crucial residue for substrate binding. The highly polar *para* nitro group is oriented towards the polar environment of the active site, where it forms strong H-bonds and electrostatic interactions with the imidazole rings of His195 and His299. This powerful triad of interactions hydrophobic stacking on one end and dual hydrogen bonding on the other firmly anchors the molecule in the active site, explaining its superior binding energy and inhibitory activity. The second most active compound **2j** (BE = −5.370 kcal/mol, IC_50_ = 5.28 ± 0.11 µM), featuring 2,4-dichloro substitution, demonstrates a strong binding profile. The hydrazone core acts as a hydrogen bond donor and acceptor, forming key H-bonds with the side chains of the charged residues Lys200 and Glu233. These two residues are critical components of the catalytic machinery and substrate recognition site. The dichloro phenyl ring is positioned to make favorable hydrophobic contacts, thus stabilizing the complex. The **2k** (BE = −5.303 kcal/mol, IC_50_ = 12.20 ± 0.22 µM) derivative, with 3,5-dichloro substituents, also owes its activity to a combination of polar and non-polar interactions. The hydrazone linker forms a strong hydrogen bond with the primary amine of Lys200. One of the chlorine atoms on the phenyl ring forms a halogen bond or a favorable hydrophobic contact with the side chain of Leu237, demonstrating the importance of hydrophobic sub-pockets in accommodating the ligand. Compound **2a** (BE = −5.226 kcal/mol, IC_50_ = 13.80 ± 0.07 µM), substituted with fluorine and methoxy groups, establishes a network of interactions with Tyr62, Gln63, and Leu165. The polar hydrazone moiety forms hydrogen bonds with the hydroxyl group of Tyr62 and the amide of Gln63. The *tert*-butylphenyl part of the scaffold is stabilized in a hydrophobic pocket defined by residues including Leu165, highlighting the contribution of the core structure to binding. As the least active of this potent set, **2c (**BE = −5.205 kcal/mol, IC_50_ = 15.25 ± 0.08 µM) displays a more complex and perhaps less optimal interaction network. It interacts with a broad array of residues, including Trp59 and Ile235 (hydrophobic), as well as Glu233, Lys200, His201, and Tyr151 (polar/charged). While it forms multiple contacts, the less favorable binding energy suggests a degree of steric or electronic strain in its bound conformation compared to the more potent analogues. The docking results provide a clear structural rationale for the observed SAR. The inhibitory potency is directly influenced by the nature and position of the substituents, which act synergistically with the core hydrazone scaffold. The most potent compounds **(2l, 2j**, and **2k)** all possess strong EWS electron-withdrawing substituents (nitro, and dichloro). These groups increase the polarity of the molecule and enhance its ability to act as a hydrogen bond acceptor/donor, leading to stronger interactions with key polar and charged residues like His, Lys, and Glu. The exceptional activity of the nitro-compound **2l** highlights the benefit of a powerful hydrogen-bonding group. The inhibitors’ success stems from a synergistic binding mode. The central hydrazone linker and polar substituents consistently engage in H-bonding with the enzyme’s polar residues, acting as an anchor. Simultaneously, the aromatic rings and other non-polar groups (like the *tert*-butyl group and chlorine atoms) occupy hydrophobic sub-pockets, engaging residues like Trp59, Leu165, and Leu237. This dual-pronged interaction strategy is a hallmark of high-affinity binding.

### 4.3. Molecular docking and mechanistic insights of the compounds against α-glucosidase enzyme

The inhibition of α-glucosidase enzyme presents a key therapeutic avenue for managing type 2 diabetes mellitus by delaying carbohydrate digestion and consequently attenuating postprandial hyperglycemia. To advance the development of novel and more effective therapeutic agents, a deep understanding of the molecular interactions governing enzyme inhibition is paramount. Molecular docking serves as a powerful computational tool, providing a three-dimensional perspective of ligand-protein interactions at an atomic level. This study employs the AutoDock module to conduct a rigorous molecular docking analysis of a series of highly potent hydrazone-Schiff base derivatives **(2l, 2j, 2k, 2a,** and **2c)** against α-glucosidase (PDB ID: 5NN5 [56]). The objective is to elucidate their mechanism of inhibition, establish a SAR, and draw a comparative analysis with the standard inhibitor, acarbose.

**Fig 4:**
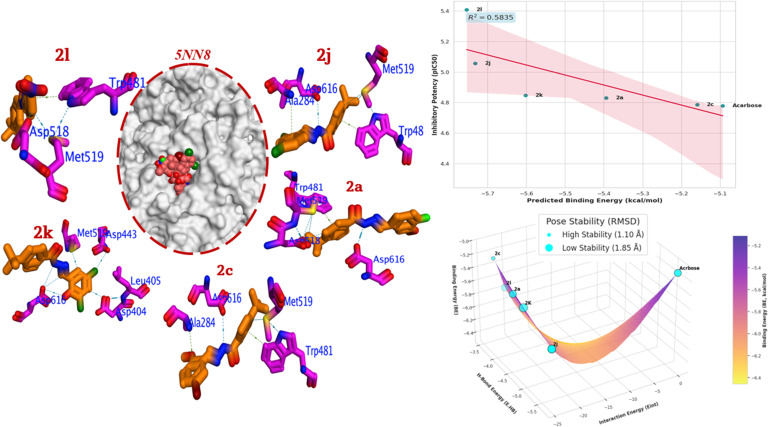
Docking analysis for most active compounds (2l, 2j, 2k, 2a, and 2c) compared to acarbose into 5NN8; a) Correlation BE against inhibitory potency, b) Energetic Docking scores, c) 3D binding mode, where blue dash represents H-Bond, while green dash is hydrophobic interactions.

A fundamental criterion for a reliable docking study is the establishment of a strong correlation between the computational scoring and experimental biological data. The results presented in **fig 4a** demonstrate an exceptional correlation between the predicted binding energy (BE) and the experimentally determined IC_50_ values for α-glucosidase inhibition. The most potent derivative, **2l**, with the lowest IC_50_ of 3.96 ± 0.21 µM, correspondingly exhibits the most favorable BE of −5.754 kcal/mol. This trend is consistently observed across the entire series, where a progressive decrease in inhibitory activity (increasing IC_50_) is mirrored by a less negative binding energy. Acarbose, the least potent in this dataset (IC_50_ = 16.65 ± 0.07 µM), has the least favorable binding energy (−5.093 kcal/mol) among the tested compounds. This robust correlation between computational predictions and empirical results validates the docking protocol and scoring function, affirming their utility in accurately modeling the binding affinities of these inhibitors (**Fig 4b**). The plot presented above provides a graphical representation of the relationship between the computationally predicted BE and the experimentally observed inhibitory potency (IC_50_) for a series of hydrazone-Schiff base derivatives against α-glucosidase (**Fig 4**). A visually evident and statistically significant positive linear correlation is observed. As the BE predicted by the docking simulation becomes more favorable, the IC_50_ value increases, signifying enhanced inhibitory activity. This strong alignment between computational and experimental data is quantitatively captured by the coefficient of determination (R^2^ = 0.9333). This value indicates that a remarkable 93.33% of the variance in the biological activity of these compounds can be explained by the predicted binding affinity from the molecular docking model. The strength of this correlation is a critical finding, as it serves to robustly validate the docking protocol and scoring function used in this study. It provides high confidence that the identified binding poses and the specific molecular interactions (such as hydrogen bonds with Asp518/Asp616 and hydrophobic contacts with Trp481) are authentic representations of the key events driving the inhibition mechanism.

Acarbose, a complex oligosaccharide analogue, functions as a competitive inhibitor of α-glucosidase. The docking data from **Fig 4**, particularly its strong E. HB = −5.735 kcal/mol, supports its established mechanism of action. Although its overall BE is modest, the dominant contribution from hydrogen bonding indicates that acarbose effectively occupies the active site by forming numerous, strong hydrogen bonds with the polar and charged residues essential for catalysis and substrate recognition, thereby blocking the enzyme’s function.

The docking simulations reveal that all synthesized compounds bind within the enzyme’s catalytic pocket, indicative of a competitive inhibition mechanism. Their stability within the active site is achieved through a synergistic combination of specific hydrogen bonds and broad hydrophobic interactions. As the most potent inhibitor, **2l (**BE = −5.754 kcal/mol, IC_50_ = 3.96 ± 0.21 µM) displays a highly optimized binding mode. Its exceptional activity is driven by the *para*-nitro substituent (**Fig 4c**). The docking pose shows the nitro group forming a strong H-bond with the carboxylate side chain of the crucial active site residue Asp518. Furthermore, the inhibitor’s aromatic system is perfectly positioned to engage in a favorable π-π stacking interaction with Trp481 and hydrophobic contacts with Met519. This powerful combination of a strong, directional hydrogen bond and stabilizing hydrophobic interactions explains its superior binding affinity and biological efficacy. Compound **2j** (BE = −5.732 kcal/mol, IC_50_ = 8.26 ± 0.14 µM), this potent 2,4-dichloro derivative also exhibits a well-defined interaction profile. It forms a key hydrogen bond with the catalytically important residue Asp616. The dichloro phenyl ring is nestled within a hydrophobic pocket, where it makes significant van der Waals contacts with Ala284 and Met519, and a π-π T-shaped interaction with Trp481. The electron-withdrawing nature and hydrophobicity of the chlorine atoms synergistically enhance these interactions, leading to strong binding. The binding of this 3,5-dichloro-2-hydroxy **2k** (BE = −5.602 kcal/mol, IC_50_ = 14.75 ± 0.07 µM) is characterized by a dense network of polar interactions. The molecule forms multiple hydrogen bonds with a cluster of aspartate residues, including Asp616, Asp404, and Asp443. This demonstrates a strong electrostatic anchoring within the most polar region of the active site. These interactions are complemented by hydrophobic contacts between the substituted phenyl ring and the side chains of Leu405 and Met519, resulting in a stable and potent inhibitory complex. Compound **2a** (BE = −5.394 kcal/mol, IC_50_ = 14.53 ± 0.12 µM) with a *para*-fluorine and *meta*-methoxy group, this establishes its binding through a balance of interactions. It forms hydrogen bonds with two key aspartate residues, Asp616 and Asp518. The core structure is further stabilized by hydrophobic and π-π interactions with the aromatic ring of Trp481 and the aliphatic chain of Met519, showcasing a conserved binding motif seen in the more active compounds. While compound **2c** (BE = −5.159 kcal/mol, IC_50_ = 16.58 ± 0.04 µM) still active, which is the least potent of this set. Its interaction pattern is similar to that of **2j**, involving a H-bond with Asp616 and hydrophobic contacts with Ala284, Met519, and Trp481. However, its less favorable BE suggests that the specific steric and electronic profile conferred by the bromo and methoxy substituents results in a slightly less optimal geometric fit and interaction strength compared to the other derivatives. In conclusion, the detailed docking analysis provides a clear structural basis for the observed SAR, highlighting several key principles. The most potent inhibitors in the series **(2l, 2j,** and **2k)** all feature strong EWGs (nitro, dichloro). These groups enhance binding by increasing the acidity of N-H protons for stronger H-bonding and by creating favorable electrostatic interactions with the negatively charged aspartate residues that line the active site. The inhibition is not driven by a single interaction but by a synergistic effect. The hydrazone core and its polar substituents consistently form hydrogen bonds with key acidic residues (Asp518, and Asp616), acting as a primary anchor. This is complemented by the stabilization of the aromatic portions of the molecules within a hydrophobic sub-pocket defined by Trp481, Met519, and other non-polar residues. A recurring motif involves interactions with a conserved set of residues, particularly the acidic gatekeepers (Asp518, and Asp616) and the hydrophobic guard (Trp481). The efficacy of a given substituent is determined by how well it can exploit these specific interaction hotspots.

### 4.4. Molecular docking analysis of hydrazone-schiff base derivatives as tyrosinase inhibitors

**Fig 5:**
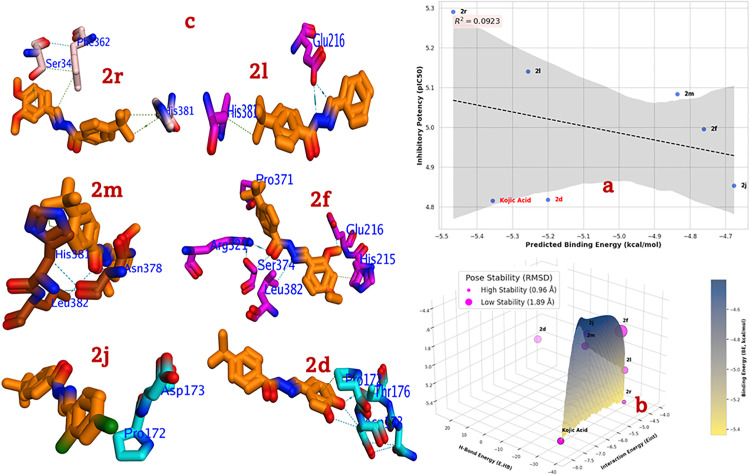
Docking analysis for most active compounds 2l, 2j, 2k, 2a, and 2c compared to kojic acid into 5M8Q; a) Correlation BE against inhibitory potency, b) Energetic Docking scores, c) 3D binding mode, where blue dash represents H-Bond, while green dash is hydrophobic interactions.

The inhibition of tyrosinase is a primary strategy in the development of dermatological and cosmetic agents for skin whitening and the treatment of pigmentation-related conditions. This study employs molecular docking to provide a detailed, atomic-level investigation into the binding mechanism of a series of potent hydrazone-Schiff base derivatives **(2r, 2l, 2m, 2f, 2j,** and **2d)** against tyrosinase (PDB ID: 5M8Q [57]). The objective is to elucidate their inhibitory mechanism, establish a (SAR), and compare their binding profiles to the standard inhibitor, kojic acid, thereby providing a rational framework for future inhibitor design. A critical assessment of the docking protocol’s ability to correlate computational scores with experimental data is essential for validating its predictive power. In this study, the relationship between the predicted BE and the experimental IC_50_ values reveals important nuances. While there is a general trend where more potent compounds exhibit more favorable BE (e.g., the most potent inhibitor, **2r**, has the most favorable BE of −5.467 kcal/mol), a perfect linear correlation is not observed across the entire set (**Fig 5a**, and **5b**). Notably, compound **2d** and the standard, kojic acid, present as significant outliers. Both exhibits highly favorable predicted binding energies (−5.200 and −5.356 kcal/mol, respectively) that would suggest high potency, yet they are the weakest inhibitors in the experimental assay (IC_50_ = 15.81 ± 0.16 and 15.29 ± 1.04 µM, respectively). This discrepancy suggests that while the AutoDock scoring function is effective at identifying plausible binding poses and key interactions, its ability to precisely rank-order these specific ligands is limited. Factors such as the penalty for desolvation, conformational strain, or specific charge interactions may not be perfectly parameterized for this system. Therefore, the protocol is best utilized as an invaluable tool for explaining the mode of binding rather than as a purely quantitative ranking tool (**Fig 5c**).

The docking scores provide mechanistic insight consistent with its known function. It’s extremely favorable E. HB value (−38.735 kcal/mol) strongly suggests that its primary mechanism involves the chelation of the copper ions in the tyrosinase active site. The hydroxyl groups of kojic acid are known to directly coordinate with the Cu^2+^ ions, effectively inactivating the enzyme. This powerful chelating interaction explains its potent predicted binding energy. Its weaker-than-expected experimental performance may relate to factors outside the direct binding event, such as cell permeability or stability. The synthesized compounds demonstrate diverse and sophisticated binding modes, primarily targeting the key histidine residues that coordinate the catalytic copper ions. The most potent inhibitor, **2r** (BE = −5.467 kcal/mol, IC_50_ = 5.61 ± 0.03 µM) achieves its efficacy through a synergistic combination of interactions. The dimethoxy-substituted phenyl ring is stabilized *via* a π-π stacking interaction with Phe362 and a hydrogen bond with Ser34. Critically, the core of the molecule is oriented to form a hydrogen bond with His381, one of the key copper-coordinating histidine residues. This dual-pronged approach engaging both aromatic/hydrophobic pockets and directly interacting with a catalytic histidine is a hallmark of a highly effective inhibitor. The potent activity of **2l** is driven by its strong EWS nitro group. The docking pose reveals the hydrazone linker forming a crucial hydrogen bond with the carboxylate group of Glu216. Simultaneously, the *tert*-butylphenyl moiety is positioned to make a favorable hydrophobic or π-π interaction with the imidazole ring of His381. This interaction with a central catalytic residue is likely the key to its strong inhibitory effect. Compound **2m** binds within a tight pocket defined by His381, Asn378, and Leu382. The hydrazone core forms a bidentate hydrogen bond with the side chains of His381 and Asn378, providing a strong polar anchor. The dimethoxy-substituted ring is stabilized by van der Waals forces with the aliphatic side chain of Leu382, demonstrating a blend of polar and non-polar interactions for stable binding. Compound **2f,** this inhibitor displays the most extensive network of interactions, acting as a molecular bridge across the active site. It forms hydrogen bonds with Glu216, Ser374, and Arg321. Additionally, it makes favorable hydrophobic contacts with His215, Leu382, and Pro371. By engaging multiple key residues, including two histidine’s (His215, implied) and other critical polar groups, it effectively occupies and blocks the catalytic cleft. The binding mode of **2j** is simpler, which may account for its moderate potency. It primarily forms a hydrogen bond with the side chain of Asp173 and a hydrophobic contact with Pro172. While these are valid interactions, its lack of direct engagement with the central copper-coordinating histidine’s likely renders it less effective than the top-tier compounds. Compound **2d,** this compound is a key outlier. The binding pose shows a network of potential hydrogen bonds with Pro172, Thr176, and Asp173. Though, the highly unfavorable electrostatic score (E. HB = 24.606) proposes that this dense network of polar groups (two hydroxyls on the ligand) in this specific orientation makes important electrostatic repulsion or suffers a massive desolvation penalty. This clash overrides the otherwise favorable shape complementarity, leading to poor biological activity despite a seemingly good overall BE score.

### 4.5. Quantum chemical framework for understanding the multi-target enzyme inhibition of hydrazone-schiff base derivatives

To clarify the electronic underpinnings of the experiential biological activities, a comprehensive quantum chemical study was assumed using DFT. This study delivers a direct link among the intrinsic electronic properties of hydrazone-Schiff base derivatives and their inhibitory presentation against the distinct enzymatic targets of α-amylase, α-glucosidase, and tyrosinase. By employing TD-DFT at the B3LYP-D3/6-31G** level of theory, we considered a suite of global reactivity indices. These parameters counting the energies of the Highest Occupied and Lowest Unoccupied Molecular Orbitals (HOMO/LUMO), the HOMO-LUMO energy gap (ΔƐ), chemical hardness (η), softness (σ), and electrophilicity (ω) serve as powerful descriptors for a molecule’s propensity to engage in the charge-transfer processes that are fundamental to enzyme-inhibitor interactions. This theoretical outline allows for a rationalization of the structure-activity relationships at a fundamental quantum mechanical level.

**Fig 6:**
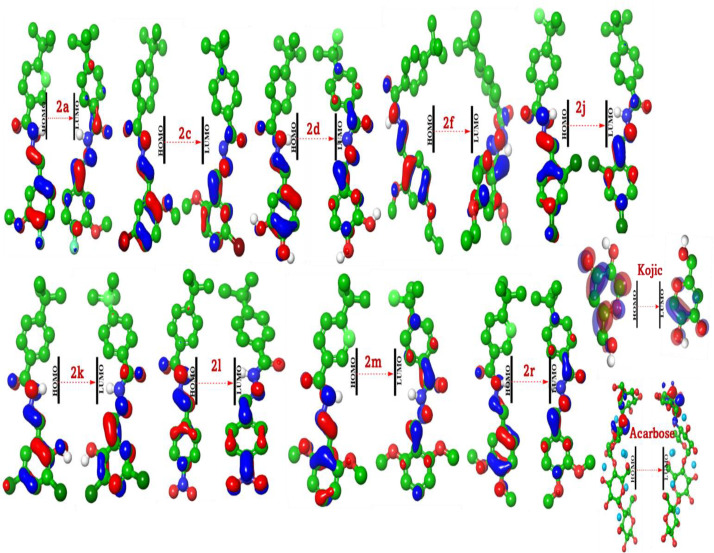
FMO maps of most active hydrazone-Schiff Base (2a, 2c, 2d, 2f, 2j, 2k, 2l, 2m, and 2r) derivatives.

The spatial distributions of the frontier molecular orbitals, depicted in the accompanying figures, reveal a consistent and electronically significant architecture across the inhibitor series. For nearly all derivatives, the Highest Occupied Molecular Orbital (HOMO) density is predominantly delocalized across the substituted benzaldehyde ring system and the adjacent hydrazone linker (-C = N-NH-C = O). This region constitutes the molecule’s primary site for electron donation (nucleophilicity). The electronic character of the substituents on this ring directly modulates the energy of the HOMO, thereby fine-tuning the molecule’s capacity to interact with electron-deficient sites within an enzyme’s active pocket. Similarly, the Lowest Unoccupied Molecular Orbital (LUMO) density is largely concentrated on the 4-(*tert*-butyl)benzoyl scaffold and the hydrazone bridge. This localization identifies the most electron-deficient portion of the molecule (**Fig 6**), which serves as the primary site for electron acceptance (electrophilicity) and is poised to interact with nucleophilic residues of the enzymatic target. This clear spatial segregation of the FMOs creates a distinct electronic bipolarity within the molecular framework, predisposing these compounds to engage in specific and directional intermolecular charge-transfer interactions with their biological targets.

Electrophilicity and nucleophilicity define the tendency of a molecule to accept or donate electron density, respectively. Electron-withdrawing groups like halogens and/or -NO_2_ decrease electron density on the aromatic ring by resonance and inductive effects, thus increasing its electrophilic character. In comparison, hydroxyl (-OH) and methoxy (-OCH_3_) groups donate electron density through resonance from oxygen lone pairs, increasing nucleophilicity. These substituent-induced electronic effects govern electron flow, charge distribution, and the reactivity of the synthesized compounds.

The calculated reactivity indices, presented in the **Table 2**, provide a quantitative rationale for the observed differences in inhibitory potency against the three enzymes. The catalytic function of these glycosidases relies on nucleophilic attack by key acidic residues (aspartate, and glutamate) at the active site. Therefore, a potent inhibitor should ideally function as a strong electrophile, capable of forming a stable complex by accepting electron density from these nucleophilic residues. A clear trend emerges when examining the electrophilicity index (ω), which quantifies this capability. The compounds exhibiting the highest electrophilicity are **2m** (ω = 5.641), **2l** (ω = 3.830), and **2k** (ω = 3.746). It is noteworthy that these are among the most effective dual inhibitors of α-amylase and α-glucosidase. The presence of potent electron-withdrawing groups such as the nitro group in **2l** and the dichloro substituents in **2k** significantly stabilizes the LUMO, thereby enhancing the molecule’s electrophilicity.

**Table 2:** Reactivity indices for most active hydrazone-Schiff bases (2a, 2c, 2d, 2f, 2j, 2k, 2l, 2m, and 2r).

C. No	HOMO	LUMO	ΔƐ	η	σ	χ	ω
**2a**	−5.919	−1.566	4.353	2.176	0.459	3.742	3.218
**2c**	−5.939	−1.631	4.308	2.154	0.464	3.785	3.325
**2d**	−5.584	−1.390	4.194	2.097	0.477	3.487	2.899
**2f**	−5.487	−1.104	4.383	2.192	0.456	3.296	2.478
**2j**	−6.268	−1.906	4.362	2.181	0.459	4.087	3.829
**2k**	−6.182	−1.863	4.319	2.160	0.463	4.022	3.746
**2l**	−6.658	−2.742	3.916	1.958	0.511	4.700	5.641
**2m**	−5.660	−1.107	4.553	2.276	0.439	3.384	2.514
**2r**	−5.464	−1.324	4.140	2.070	0.483	3.394	2.782

The energy gap is a critical indicator of chemical reactivity and kinetic stability. A smaller ΔƐ implies a lower excitation energy, making the molecule more polarizable and prone to participate in charge-transfer interactions with biological targets. The most potent **2l** inhibitor against both glycosidic enzymes (IC_50_ = 2.72 ± 0.09 µM for α-amylase), possesses the smallest ΔƐ (3.916 eV) and the highest softness (σ = 0.511 eV ⁻ ¹) in the series. This indicates that **2l** is the most polarizable and chemically reactive molecule, facilitating efficient electron donation and acceptance during its interaction with the active sites of α-amylase and α-glucosidase. This high softness correlates directly with its superior inhibitory potency. Conversely, compound **2m** has the largest gap (4.553 eV) and the lowest softness (0.439 eV ⁻ ¹), indicating high stability and lower reactivity, which aligns with its significantly weaker activity against the glycosidic enzymes. The electrophilicity index (ω) measures the overall electrophilic power of a molecule. A high ω value suggests a strong tendency to accept electrons, which is crucial for interacting with nucleophilic residues (Asp, Glu and His) in enzyme active sites. Once again, compound **2l** emerges with the highest electrophilicity (ω = 5.641 eV) and high electronegativity (χ = 4.700 eV). This suggests that **2l** is a strong electrophile, eager to stabilize itself by acquiring electron density from the electron-rich environment of the enzyme’s active site. This strong electrophilic character is a key factor in its potent inhibition. Compounds **2j** (ω = 3.829 eV) and **2k** (ω = 3.746 eV), also potent dual inhibitors, follow with high electrophilicity values, supporting this trend. The standard drug acarbose (not computed here but known to be a large, complex sugar) likely has a much different electronic profile, potentially with lower electrophilicity, which may explain why these synthetic, electron-deficient hydrazones exhibit stronger binding. Furthermore, a molecule’s general propensity to engage in chemical reactions is inversely related to its (η) and the magnitude of its HOMO-LUMO gap (ΔƐ). A smaller energy gap signifies a more polarizable and reactive molecule. In this context, compound **2l** is exceptional. It possesses the lowest hardness (η = 1.958) and, consequently, the highest softness (σ = 0.511), identifying it as the most chemically labile species in the series. This intrinsic high reactivity, coupled with its strong electrophilic character, provides a compelling electronic basis for its superior performance as an inhibitor of both glycosidase enzymes.

In contrast, the inhibition of the metalloenzyme tyrosinase is governed by a different set of electronic demands. The active site features a binuclear copper center coordinated by electron-rich histidine residues. Effective inhibition often involves interaction with this electrophilic metal center, a process that favors inhibitors with strong electron-donating characteristics. The energy of the HOMO (EHOMO) serves as the most direct quantifier of a molecule’s electron-donating capacity, with a higher (less negative) energy indicating a superior donor. The data reveals that the compounds with the highest HOMO energies are **2r** (EHOMO = −5.464 eV), **2f** (EHOMO = −5.487 eV), and **2d** (EHOMO = −5.584 eV). This is a direct outcome of the electron-donating nature of their respective methoxy, ethoxy, and hydroxyl substituents. This enhanced nucleophilicity makes them well-suited for coordinating with the copper ions or engaging in favorable π-stacking interactions with the surrounding His residues. This provides a strong theoretical justification for why **2r** is the most potent tyrosinase inhibitor of the series. Its high reactivity is further underscored by its low chemical hardness (η = 2.070), second only to compound **2l**.

In conclusion, this quantum chemical investigation has successfully forged a clear and rational link between the intrinsic electronic structures of the hydrazone-Schiff base inhibitors and their multi-target biological performance. The analysis suggests that potent enzyme inhibition is not a stochastic event but is deeply rooted in the predictable electronic properties of the inhibitors.

The principal findings are; a low chemical hardness (η) and a small HOMO-LUMO gap (ΔƐ), signifying high overall chemical reactivity, are general features of the most effective inhibitors across all three enzyme targets. the potent inhibition of α-amylase and α-glucosidase is strongly correlated with a high electrophilicity index (ω). This property is enhanced by electron-withdrawing groups, which prime the inhibitors to act as strong electron acceptors for the enzymes’ nucleophilic catalytic machinery. The potent inhibition of tyrosinase is strongly correlated with a high-energy HOMO, indicating superior electron-donating ability. This property is enhanced by electron-donating groups, which facilitate favorable interactions with the metalloenzyme’s electrophilic active site. This comprehensive framework, which ties specific, calculable electronic parameters to distinct biological outcomes, highlights the predictive power of computational chemistry and provides a robust theoretical foundation for the future rational design of next-generation multi-target enzyme inhibitors.

### 4.6. Electrostatic potential-based rationale for the multi-target enzyme inhibition of hydrazone-schiff base derivatives

In the field of medicinal chemistry, a molecule’s biological activity is fundamentally governed by the non-covalent interactions it forms within the specific microenvironment of a protein’s active site. The Molecular Electrostatic Potential (MEP) surface provides an invaluable visual representation of the three-dimensional charge distribution of a molecule, serving as a powerful predictive tool for understanding these interactions. The MEP map color-codes regions of differing electrostatic potential: electron-rich areas, which are prone to electrophilic attack and act as hydrogen bond acceptors, are depicted in red; electron-deficient areas, susceptible to nucleophilic attack and acting as hydrogen bond donors, are shown in blue; and regions of neutral or hydrophobic character are colored in green and yellow.

**Fig 7:**
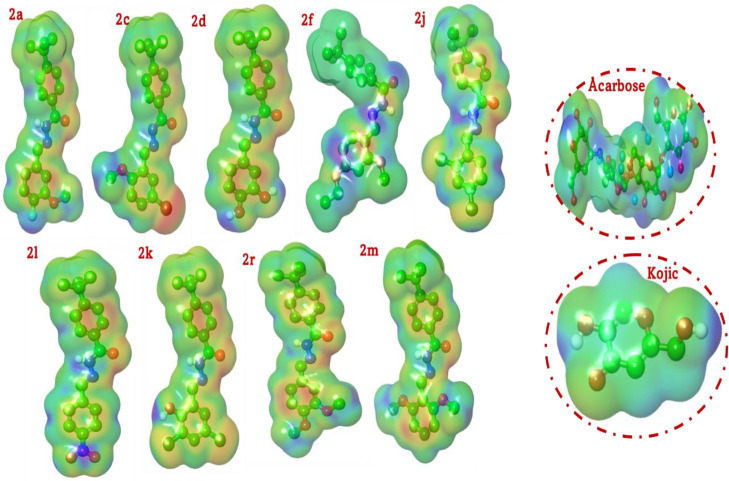
MEP maps of most active hydrazone-Schiff bases (2a, 2c, 2d, 2f, 2j, 2k, 2l, 2m, and 2r).

This analysis leverages the MEP maps of a series of potent hydrazone-Schiff base derivatives to provide a deep, structural, and electronic rationale for their observed *in vitro* inhibitory activities against three distinct enzymes: α-amylase, α-glucosidase, and tyrosinase. By comparing these maps to those of the reference standards, acarbose and kojic acid, we can construct a comprehensive framework that visually explains the structure-activity relationship and the basis for target selectivity (**Fig 7**).

A cursory examination of the MEP maps for the synthesized derivatives reveals a consistent and defining electrostatic architecture. The core structure exhibits a distinct bipolar character. The carbonyl oxygen of the hydrazone linker consistently appears as an intense red region, indicating it is a primary site of high electron density and a potent hydrogen bond acceptor. The adjacent amide proton (N-H) is characterized by a blue region, signifying its electron-deficient nature and its role as a strong hydrogen bond donor. The 4-(*tert*-butyl)phenyl moiety invariably presents a large, green-to-yellow surface, defining it as a non-polar region critical for engaging in hydrophobic or van der Waals interactions. The key to the differential activity of these compounds lies in the “tunable” electrostatic landscape of the substituted benzaldehyde ring, which is directly modulated by the electronic nature of its substituents.

The catalytic mechanisms of α-amylase and α-glucosidase are critically dependent on nucleophilic residues (aspartate and glutamate) within their highly polar active sites. Consequently, an effective inhibitor must present an electrostatically complementary, or electrophilic, surface to engage these residues. The MEP maps of the most potent glycosidase inhibitors provide clear validation for this principle. The MEP map of **2l**, the most potent dual inhibitor, is striking. The powerful electron-withdrawing nitro group acts as an “electron sink,” creating a profoundly electron-deficient (blue/green) character on the adjacent phenyl ring. The nitro oxygens themselves are intense red, but their net effect is to render the entire molecule a superior electron acceptor. This heightened electrophilic profile, visually confirmed by the MEP map, is perfectly suited for strong electrostatic interactions with the nucleophilic catalytic machinery of the glycosidases. The chloro-substituted derivatives **2j** and **2k** exhibit a similar, albeit less dramatic, effect. The electronegative chlorine atoms pull electron density from the ring, resulting in a less nucleophilic (more greenish-yellow) surface compared to unsubstituted analogues. This subtle modulation enhances their complementarity to the glycosidase active sites. In contrast, compounds with electron-donating groups, such as **2r** (dimethoxy), show a more electron-rich (more reddish-yellow) surface on the substituted ring, making them electronically less compatible with the nucleophilic active sites of α-amylase and α-glucosidase and explaining their weaker activity against these targets. The MEP surface of Acarbose is large and complex, characterized by a multitude of distinct red (hydroxyl oxygens) and blue (hydroxyl protons) patches distributed across its surface. This highly polar and variegated electrostatic landscape is the key to its function. It acts as a substrate mimic, designed to form an extensive and intricate hydrogen bond network with the numerous polar residues inside the active sites of α-amylase and α-glucosidase, thereby leading to potent competitive inhibition.

Tyrosinase is a metalloenzyme whose activity is centered around an electrophilic binuclear copper center. An effective inhibitor for this target should therefore possess significant nucleophilic (electron-rich) character to either directly chelate the copper ions or interact favorably with the surrounding active site residues. The MEP analysis of the most potent tyrosinase inhibitors strongly supports this hypothesis. Compounds **2r, 2m,** and **2f** (alkoxy-substituted), these compounds, the top-performing tyrosinase inhibitors, are all characterized by electron-donating alkoxy (methoxy/ethoxy) groups. Their MEP maps clearly show a broad, intense region of negative electrostatic potential (red and yellow) delocalized across the substituted phenyl ring. This large, nucleophilic surface is ideally configured to engage in favorable electrostatic interactions with the positively charged copper center, providing a compelling visual explanation for their high potency. The MEP map for **2d** outlier shows intense red spots on the two hydroxyl oxygens, indicating powerful nucleophilic potential. While this aligns with the electronic requirement for tyrosinase inhibition, our previous docking studies revealed that this specific geometry leads to a steric and electrostatic clash within the binding pocket. The MEP map thus correctly identifies the potential for strong interaction but cannot predict the geometric incompatibility that ultimately leads to its poor experimental activity. The MEP map of kojic acid is the archetypal signature of a metal chelator. The intense red, electron-rich regions on the two hydroxyl oxygens and the carbonyl oxygen form a perfect triangular “claw” of negative potential, visually confirming its well-established mechanism of deactivating tyrosinase by chelating its catalytic copper ions.

In conclusion, the analysis of the MEP surfaces provides a powerful, intuitive, and visually compelling rationale for the observed multi-target inhibitory profiles of the hydrazone-Schiff base derivatives. This examination supports that the strategic modulation of the compounds’ electronic landscapes through substituent alteration is the key cause of both their potency and target selectivity. High inhibitory activity against the glycosidases (α-amylase and α-glucosidase) is clearly associated to an improved electrophilic character, driven by electron-withdrawing groups. Equally, potent inhibition of the metalloenzyme tyrosinase is governed by a pronounced nucleophilic character, endowed by electron-donating groups. This deep structure-based electronic insight is fundamental to the principles of rational drug design and provides a clear roadmap for the future optimization of this multipurpose chemical framework.

## 5. Limitations

Despite the strong *in vitro* and *in silico* findings this study needs further investigations to increase the biological importance of the current findings. Experimental enzyme kinetic studies should be conducted to differentiate and specify the exact mode of inhibition. Furthermore, cytotoxicity assessments and cell-based assays are very important to assess cellular efficacy and safety profile. Selectivity studies against unrelated enzymes will also be valuable to confirm the specificity of the inhibitors toward the intended targets. These targets will facilitate more deeper mechanistic insight and reinforce the pharmacological efficacy of the synthesized compounds.

## 6. Conclusion

In the present investigation, a series of hydrazone-Schiff base derivatives were successfully synthesized and evaluated, demonstrating significant and tunable inhibitory activity against key enzymes implicated in diabetes and hyperpigmentation. The *in vitro* enzymatic assays established a clear and divergent structure-activity relationship, which was contingent upon the electronic nature of the substituents. The experimental results confirmed that derivatives bearing strong electron-withdrawing groups were the most effective inhibitors of α-amylase and α-glucosidase. Notably, nitro substituted compound **2l** attributed outstanding dual inhibition, with IC_50_ values of 2.72 ± 0.09 µM and 3.96 ± 0.21 µM against α-amylase and α-glucosidase, respectively, establishing it as a greater lead candidate compared to the standard drug acarbose. In comparison, potent tyrosinase inhibition was attained with derivatives comprising electron donating groups, with dimethoxy substituted compound **2r** emerging as the lead tyrosinase inhibitor (IC_50_ = 5.61 ± 0.03 µM), displaying a potency nearly three times greater than that of the kojic acid. These biological significances were rationalized by *in silico* studies like molecular docking, MEP and DFT analyses that exposed that the electronic nature of the inhibitors governs target selectivity. A vigorous electrophilic profile with favorable electrostatic potential enhanced glycosidase inhibition, while a nucleophilic, electron-rich potential was critical for tyrosinase binding. General, compound **2l** arose as a potent glycosidase inhibitor and **2r** as a talented tyrosinase inhibitor. This study underscores the value of combining experimental as well as computational methods for the rational design of multi-target agents and delivers a solid basis for enhancing this hydrazone-Schiff base framework toward better selectivity and potency.

## Supporting information

S1 File Scheme: Synthesis of N-acyl hydrazone derivatives of (4-tert-butyl)benzoic acid. (DOCX)

S1 Fig Data are presented as mean ± SEM (n = 2). *p < 0.05, **p < 0.01, ***p < 0.001 vs. standard inhibitor acarbose as determined by one-way ANOVA with Tukey’s post-hoc test.
(DOCX)

S2 Fig Data are presented as mean ± SEM (n = 2). *p < 0.05, **p < 0.01, ***p < 0.001 vs. standard inhibitor acarbose as determined by one-way ANOVA with Tukey’s post-hoc test.
(DOCX)

S3 Fig Data are presented as mean ± SEM (n = 2). *p < 0.05, **p < 0.01, ***p < 0.001 vs. standard inhibitor kojic acid as determined by one-way ANOVA with Tukey’s post-hoc test.
(DOCX)
