# Supplementary material for: Hydrazone-schiff base derivatives of 4-(tert-butyl)benzoic acid as potent enzyme inhibitors: In vitro α-amylase, α-glucosidase, tyrosinase inhibition and computational studies
Source: PLoS One. 2026 May 11;21(5):e0348140. doi: 10.1371/journal.pone.0348140 (PMC13160446; doi:10.1371/journal.pone.0348140)
Supplement: S1 File — (DOCX) [file pone.0348140.s001.docx]

**Scheme-S1:** Synthesis of *N*-acyl hydrazone derivatives of 4-(tert-butyl)benzoic acid.
