# Supplementary material for: Hydrazone-schiff base derivatives of 4-(tert-butyl)benzoic acid as potent enzyme inhibitors: In vitro α-amylase, α-glucosidase, tyrosinase inhibition and computational studies
Source: PLoS One. 2026 May 11;21(5):e0348140. doi: 10.1371/journal.pone.0348140 (PMC13160446; doi:10.1371/journal.pone.0348140)
Supplement: S1 Fig — *p < 0.05, **p < 0.01, ***p < 0.001 vs. standard inhibitor acarbose as determined by one-way ANOVA with Tukey’s post-hoc test. (DOCX) [file pone.0348140.s002.docx]

**Fig S1:** Data are presented as mean ± SEM (n=2). *p < 0.05, **p < 0.01, ***p < 0.001 vs. standard inhibitor acarbose as determined by one-way ANOVA with Tukey's post-hoc test.
